# Assessing understandings of substance use disorders among Norwegian treatment professionals, patients and the general public

**DOI:** 10.1186/s12913-016-1306-9

**Published:** 2016-02-13

**Authors:** John-Kåre Vederhus, Thomas Clausen, Keith Humphreys

**Affiliations:** Addiction Unit, Sørlandet Hospital HF, P.b. 416, 4604 Kristiansand, Norway; Norwegian Center for Addiction Research, University of Oslo, Oslo, Norway; Veterans Health Administration, Palo Alto, California USA; Stanford University School of Medicine, Stanford, California USA

**Keywords:** Substance-related disorders, Attitudes of health personnel, Professional-patient relations, Patient-centered care, Health services research, Alcoholics anonymous

## Abstract

**Background:**

Beliefs about substance use disorder (SUD) shape how patients, treatment professionals and the general public view addiction and its treatment. A U.S. developed scale exists to assess such beliefs, but it has never been tested in Norway nor normed on any general population sample.

**Methods:**

The *Short Understanding of Substance Abuse Scale* (SUSS) was translated from English to Norwegian and used to assess beliefs about the nature of addiction among addiction treatment professionals (*N* = 291), patients with SUDs (*N* = 133) and respondents from the general public (*N* = 216). The disease and psychosocial model subscales of the SUSS were examined with a multigroup factor analysis to confirm that the constructs were invariant across the studied groups. We also controlled for demographic covariates in a multiple indicator multiple cause model.

**Results:**

The multigroup confirmatory factor analysis of the SUSS yielded a partial scalar invariant model and thus, we were able to compare latent means between groups. In unadjusted comparisons, patients and the general public reported significantly higher endorsement of disease model beliefs than did professionals. However, the difference between professionals and the general public disappeared when the comparison was adjusted for covariates (i.e., age, gender, education). In both unadjusted and adjusted analyses, the general public group but not the patient group scored significantly lower than professionals on the psychosocial belief scale.

**Conclusion:**

The SUSS is useable with slight adaptations in Norwegian samples. Norwegian treatment professionals have different views of substance use disorder than do patients and the general public. This may create opportunities for dialogue and mutual learning, but also presents risk of miscommunication and distrust.

## Background

How treatment providers and treatment seekers conceptualize substance use disorders (SUD) has many practical implications [1–3], including for example whether persons with addiction are blamed for their SUD [4]. Different understandings of etiology may influence clinicians’ choice of treatment strategies (e.g., behavioral self-control training versus 12-step facilitation counseling) and the treatment goals to which the clinician and patient agree (e.g., abstinence versus controlled use) [5, 6]. Among individuals with a SUD, the understanding of addiction could potentially influence whether they seek treatment or try to solve the problem on their own [7]. The beliefs of the general public, though rarely studied, are also of interest as they may influence their willingness to support publically funded treatment services and to encourage loved ones to access them. Beliefs about SUD are thus clearly important to study in health services research, making it valuable to have reliable instruments that have been validated in multiple cultural contexts.

The *Short Understanding of Substance Abuse Scale* (SUSS) was developed in the U.S. to measure therapist and patient beliefs regarding substance dependence [3, 8]. The SUSS is a modification of Moyers and Miller’s longer alcohol-focused “Understanding of Alcoholism Scale” [2]. The SUSS assesses beliefs about illicit drugs as well as alcohol in three domains; disease model, psychosocial model and eclectic model beliefs. The *disease model* comprises beliefs that alcohol and drug dependence are chronic illnesses that can only be arrested through life-long abstinence. The disease model beliefs has its origin in the disease concept of the 12-step treatment model [4, 9], which in turn was influenced by the 12-step program of Alcoholics Anonymous (AA) [10, 11]. The disease model beliefs have also recently been associated with the “brain disease” concept of addiction (which AA did not endorse) prominently promoted by NIDA researchers, with its emphasis on disturbed neurobiology of long-term duration [12, 13]. The *psychosocial model* beliefs reflect the view that substance misuse is a product of psychological and social factors (e.g., cultural influences and family environment) [2, 8, 14]. The meaning of the third scale, *the eclectic model* is less clear, but items loading on this factor reject the idea that addiction is a homogeneous entity with a single etiology [2]. Research in both the U.S. and Switzerland showed that this subscale had mediocre psychometric properties, leading to the recommendation to use only the first two scales [2, 15].

In previous studies with the SUSS among professionals, higher age was associated with greater endorsement of the disease model beliefs [1]. Higher educational level was associated with less endorsement of disease model beliefs and higher endorsement of psychosocial model beliefs [1]. A study conducted in outpatient treatment settings found that the more a practitioner’s beliefs about dependence reflect a psychosocial understanding, the more likely s/he would be to use a cognitive or motivational treatment approach [6]. Conversely, the more practitioners’ beliefs reflect a disease model understanding of dependence, the more likely they would be to use a directive or 12-step treatment approach. Similarly, there is a negative association between disease model beliefs and endorsing “moderate drinking” as a treatment goal [5]. Among patients, stronger adherence to the disease model beliefs was associated with more involvement in supportive fellowships like the 12-step groups following treatment [3]. To our knowledge, the SUSS has not been used to directly compare professionals’ beliefs to those of patients or to the general public. Neither has it been employed in Norway, a country where the 12-step disease model has been less influential than it has in the U.S. where the SUSS was originally developed [16, 17].

Before applying a scale developed in one culture to another (or indeed in a different subculture within the same society), one ought to check whether the questionnaire measures the constructs in the same way in the respective cultures. The SUSS was developed in the U.S., and had a similar but somewhat different factor structure in a Swiss German sample; one disease model item was replaced with an eclectic model item as a result of their exploratory factor analysis [15]. Thus, one needs to affirm that the measurement and the structure of the underlying constructs are invariant (equivalent) across the studied samples [18, 19]. The equality of meaning of the items is usually assumed between groups but not tested; the same is true for the intercepts of the items. Different understanding and scoring of survey responses may be due to educational level, cultural background or experiences of SUD [18]. Non-equivalence can also exist if different mode of data collection is used [20]. Thus, one should use statistical approaches to test measurement equivalence. Multigroup confirmatory factor analysis (MGCFA) is the most widely used method for such testing [18].

The aim of this study was to test the measurement equivalence of the SUSS across Norwegian groups (professionals, patients and the general public), and then examine differences between the groups regarding the understanding of substance dependence.

## Methods

### Participants and procedures

The addiction treatment professional group (hereafter referred to as ‘professionals’) participated in a cross-sectional study in the southern five counties of Health Region South East, Norway, in 2008. That study aimed to describe participants’ attitudes towards and referral practices to Twelve Step mutual help organizations. An assigned contact person in each treatment center recruited the participants and returned the below described questionnaire anonymously to the researchers. Returning the survey was considered as an implied consent. The response rate was 80 % (291 of 365 eligible respondents). Most (80 %) worked in in-patient units. The patient group was enrolled by study staff in a clinical trial at the in-patient detoxification ward at the Addiction Unit, Sørlandet Hospital in Kristiansand, Norway from September 2008 to August 2010. The participants provided written informed consent before inclusion. Of 156 eligible patients, 16 refused participation and seven did not provide answers to any question on the main instrument (see below), yielding *N* = 133 for the analysis (85 % response rate). The participants in both of these studies filled out the below described paper based questionnaire as a part of their respective study, each of which has been described in detail elsewhere [16, 17, 21, 22]. The Regional Ethics Committee of the South-East Health Region, Norway, approved both studies. No incentives were offered to participants. The Addiction Unit at the Sørlandet Hospital granted permission to use the data from these previous studies. The general population sample (*N* = 216) was recruited via Facebook, with a link to an online survey posted on several Facebook groups or spread via Facebook contacts of the first author. The data collection was carried out from October 15 to October 20, in 2014. The inclusion criteria were that respondents should be > 18 years old and not employed in the addiction treatment or the psychiatric services field. Participants were asked to verify these criteria before continuing the online survey. Data collection was anonymous, thus, ethical approval and consent was deemed unnecessary according to national regulations [23]. Nevertheless, replying to the survey was considered as an implied consent.

### Measures

The English SUSS version was translated to Norwegian with a standard procedure (two forward and two backward translations), in collaboration with the questionnaire’s lead developer (the third author of the present paper) [24]. The SUSS consists of seven statements for the disease model beliefs and five statements for the psychosocial model beliefs subscales (Table 1), which were found to cluster together in the only European psychometric study of the instrument [15]. We did not use the eclectic model subscale in this study, as recommended [2, 15]. Respondents rated the statements on a 5-point Likert scale ranging from the strongest disagreement (score 0) to the strongest agreement (score 4) [8].

**Table 1:** Short understanding of substance abuse scale (SUSS)^a^

Disease model beliefs	
*Q1_D	- Every alcoholic and addict must accept that he or she is powerless over alcohol and drugs, and can never drink or use drugs again
Q2_D	- Every alcoholic or addict is one drink or one hit away from a total relapse
Q5_D	- Once a person is an alcoholic or an addict, he or she will always be an alcoholic or an addict
*Q7_D	- Usually if alcoholics and addicts fail to recover in AA / NA or treatment, it is because they are unmotivated and in denial
Q8_D	- If an alcoholic or addict is sober or straight for five years, then starts drinking or using drugs again, he or she is right back where he or she left off in the development of the disease
Q11_D	- There are only two possibilities for an alcoholic or drug addict – permanent abstinence or death
Q12_D	- If an alcoholic has a drink, or if an addict takes a hit, they lose control and are unable to stop from getting drunk or high
Psychosocial model beliefs	
Q3_P	- The society or culture in which on grows up has a significant influence on whether or not one becomes an alcoholic or an addict
Q4_P	- A person’s environment plays an important role in determining whether he or she develops alcoholism or drug addiction
*Q6_P	- Alcoholism and drug addiction are caused, in part, by growing up in a dysfunctional family
Q9_P	- Alcoholism and drug addiction are caused, in part, by what one learns about alcohol and drugs and the drinking/drug use patterns of one’s family and peers
*Q10_P	- A person can develop alcoholism or drug addiction because of underlying psychological problems

Note:

^a^Humphreys K, Greenbaum MA, Noke JM, Finney JW: Reliability, validity, and normative data for a short version of the Understanding of Alcoholism Scale. *Psychol Addict Behav* 1996, 10(1):38–44

*Marked questions were excluded in the multigroup confirmatory factor analysis and multiple indicator multiple cause model analysis due to the development of the configural (baseline) model

### Statistical analyses

Demographics are presented with descriptive statistics, as also were the observed mean and sum scores of the SUSS subscales. We conducted a simultaneous MGCFA according to standard procedure (the two constructs under study were tested simultaneously) [25, 26]. MGCFA is a special case of the structural equation modeling approach and is based on the idea that theoretical concepts are not directly observable, but can be inferred from observed indicators (i.e., questions) that reflect the underlying construct (e.g., disease model beliefs) [27]. The analysis accounts for both random and nonrandom measurement error [28]. Analyzes were undertaken with the software program Mplus, version 7.3, and we used the maximum likelihood estimation with robust standard errors (MLR) [29]. To handle missing values, the default procedure in Mplus, full information maximum likelihood was used.

First, baseline (configural) models for each group were established. We understood and analyzed the questions as reflective indicators of their respective construct, which means that they are exchangeable; i.e., one can reduce the number of items if necessary, without changing the content of the underlying latent variable [25]. Thus, to find the most parsimonious model, problematic items were removed if they did not work well in the analysis [e.g., had low factor loadings (<0.4) or high error correlation with other questions] [25, 30]. As global goodness of fit criteria we used the Root Mean Square Error of Approximation (RMSEA) (cut-off value for a good model fit < .06 and an acceptable fit .06–.08), and the Comparative Fit Index (CFI) (cut off value for a good model fit > .95 and an acceptable fit between .90 and .95) [31, 32].

Proceeding to the multigroup analysis, the relations between the latent variables and their respective indicators across groups were compared [28]. To be able to compare latent means, strong measurement invariance (scalar equivalence) is needed [33]. Scalar equivalence implies that the measurement scales do not only have the same factor structure (i.e., configural equivalence) and equal factor loadings (i.e., metric equivalence) across groups, but also invariant intercepts, i.e., equivalent origin on the scales in the different groups [18]. We used the new and simplified feature in Mplus 7 to test for invariance and included the syntax ‘MODEL = Configural Metric Scalar’ in the ‘Analysis’ subsection. This procedure implements cross-group equality constraints on measurement parameters and compares more restricted models with less restricted models (nested models) [29]. Chi-square difference tests between the nested models are used in which the difference in chi-square value (∆*χ*2) relative to the change in degrees of freedom (∆*df*) are evaluated, as are also the changes in CFI (∆CFI) [29]. A non-significant ∆*χ*^2^ value or a ∆CFI smaller than or equal to −0.01 indicate that constraining the parameters does not significantly worsen the fit of the model, and the null hypothesis of measurement invariance can be retained [34]. If our model showed not to be scalar invariant, intercepts of the non-invariant variables would be freed to assume partial scalar equivalence, which is still considered sufficient for comparing latent means if at least two intercepts and loadings per construct are invariant [33]. Differences in latent means were examined with the ‘professionals’ as the reference groups. The significance level was set at *p* < 0.05.

In a next step we specified a multiple indicators multiple causes model (MIMIC) to control the latent mean differences between the three groups for differences in the distribution of age, gender and education between the groups [35, 36]. For the specification and test of the model, it was necessary to transform group membership to dummy variables. The professional group was chosen as a reference group. In addition to the two dummy variables representing patients and general public, age, education and gender was added as covariates. The regression coefficients of the dummy variables (groups) can be interpreted as mean differences [36].

## Results

**Fig. 1:**
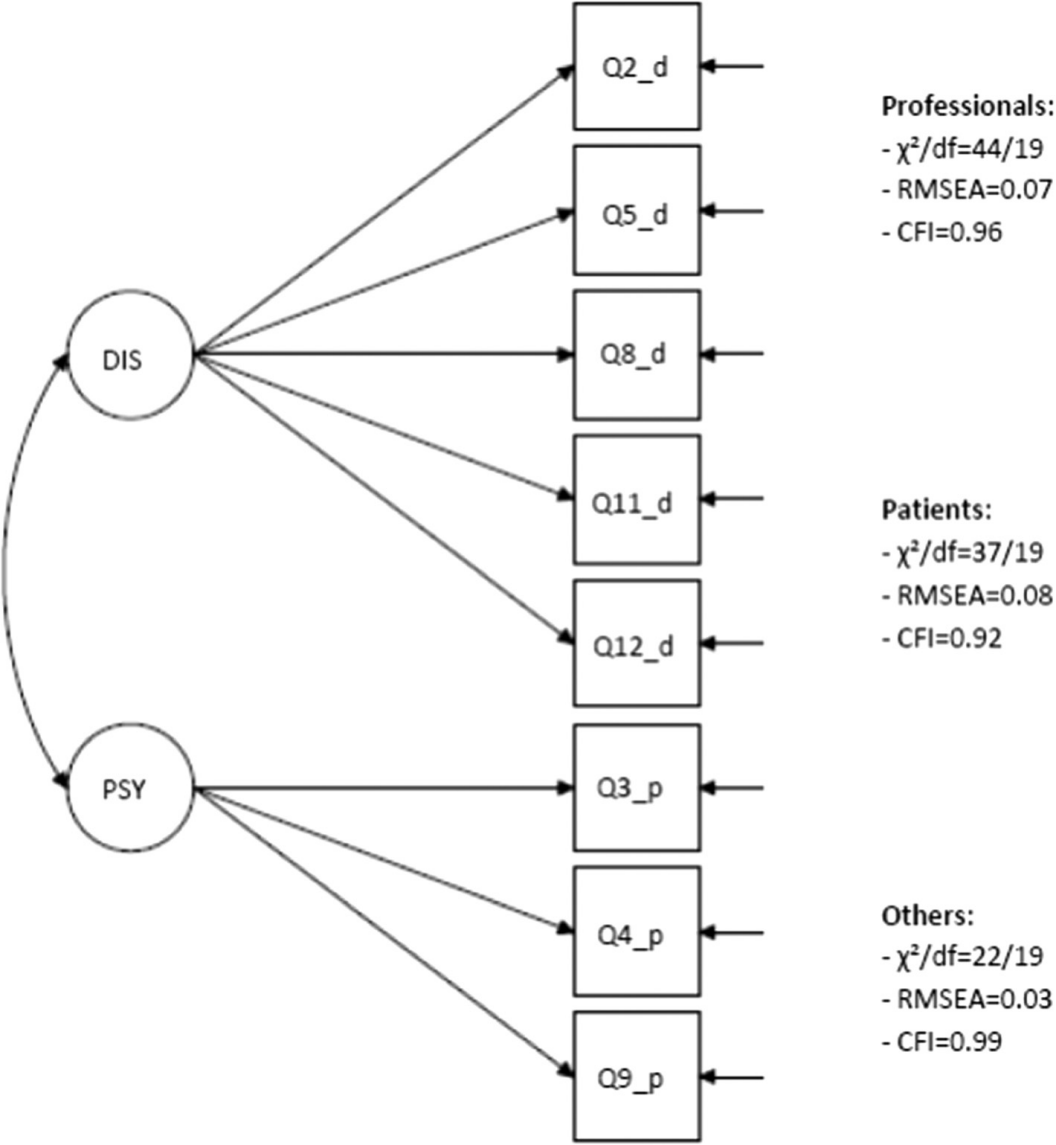
Baseline model for the multigroup confirmatory factor analysis of the *Short Understanding of Substance Abuse Scale* (SUSS) in the three studied groups: professionals, patients and general public, *N* = 640 participants. Notes: − DIS = disease model beliefs - PSY = psychosocial model beliefs - Q2_d, Q5_d, Q8_d, Q11_d, Q12_d = indicators for the disease model beliefs, see Table 1 for question wording - Q3_p, Q4_p, Q9_*p* = indicators for the psychosocial model beliefs, see Table 1 for question wording

The covariance coverage exceeded 98.8 % on all items, which is excellent. Demographics and observed scorings on the SUSS are shown in Table 2. In the following section we refer to question numbers of the SUSS (Table 1). In the psychosocial belief subscale, question 10 had a very low loading (β = 0.16) and a non-significant R^2^ (0.02, *p* = 0.486) in the ‘patient’ sample. Similarly, question 6 had a low loading (β = 0.34) and non-significant R^2^ (0.12, *p* = 0.06) in the general population sample. This implies no or a very weak relationship between construct and indicator [25]. Thus, these items were removed from the baseline model and the multiple group analysis. We also chose to remove two items in the disease belief subscale; question 7 because of low factor loadings in two of the groups (∼0.4) and question 1 due to a high error correlation with question 2 in the general public group. The modification indices (MI) indicated that a chi-Square reduction of 24 (standardized parameter change) was expected if these residuals were allowed to correlate. The high error correlation can be explained by the very similar wording and content of these two questions. After these adjustments of the basic model, the baseline models for the different groups had acceptable goodness-of-fit measures, and we could use the same baseline model for each group (Fig. 1).

**Table 2:** Demographics and observed scorings on the full *Short Understanding of Substance Abuse Scale* (SUSS) for *N* = 640 participants^a^

	Professionals *N* = 291	Patients *N* = 133	General public *N* = 216
Age (years)	45 (10)	41 (14)	43 (15)
Gender (female %)	72	34	62
Educational length (years)	15.6 (2.5)	11.2 (2.3)	14.2 (2.7)
Disease model belief; sum score^b^	15.8 (5.9)	21.4 (5.7)	16.9 (5.9)
Psychosocial model belief; sum score^c^	13.0 (2.5)	13.2 (3.5)	12.0 (3.1)
Disease model belief; mean score^d^	2.2 (0.9)	3.0 (0.9)	2.3 (0.9)
Psychosocial model belief; mean score^e^	2.6 (0.6)	2.6 (0.8)	2.4 (0.8)

Notes:

^a^Variables are shown as mean (SD)

^b^Sum score of seven questions; 0–28 scale

^c^Sum score of five questions; 0–20 scale

^d^Mean of seven questions, score 0–4

^e^Mean of five questions, score 0–4

The multigroup analysis indicated that the model as a whole was metric equivalent; the corrected Chi-square difference test between the configural and metric model was non-significant (∆*χ*^2^/∆*df* difference = 17/12, *p* = 0.17), and the ∆CFI was < −0.01 (Table 3). This means that the factor loading between the groups did not differ according to both criteria and the meaning of the constructs can be compared over the three groups. However, the model was not scalar equivalent as the corrected difference test between the metric and the scalar model was highly significant (∆*χ*^2^/∆*df* = 66/12, *p* < 0.001), and the ∆CFI was > −0.01. As expected, the multigroup scalar model only had acceptable fit indices (RMSEA = 0.08, CFI = 0.91); the MIs indicated that the intercept of question 5 was non-invariant. When this intercept was freed (i.e., a partial scalar model), the model showed a good fit (RMSEA = 0.06, CFI = 0.95), and the ∆CFI was −0.01 compared with the configural model (Table 3). This implies that we reached full metric invariance and partial scalar invariance, which is sufficient for comparing latent means [19].

**Table 3:** Multigroup confirmatory factor analysis results of the measurement invariance tests across the three groups (professionals, patients and general public, *N* = 640)

	*χ* ^2^	*df*	RMSEA^a^	CFI^b^
Configural model	104	57	0.06	0.96
Metric model	121	69	0.06	0.96
Scalar model	187	81	0.08	0.91
Partial scalar model	143	79	0.06	0.95

Notes:

^a^RMSEA = Root Mean Square Error of Approximation

^b^CFI = Comparative Fit Index

The differences in latent means between groups can be seen in Table 4. The professionals served as the reference group. Compared to the professionals, the patients had a significantly ∼ 1 point higher mean score on the disease model belief scale. The psychosocial belief score was not significantly different. The general population group also had a significantly higher disease model belief score compare to the reference group. However, the nominal distance was much smaller with a 0.25 point higher mean score. The psychosocial belief score was significantly lower than the professionals, but again, the nominal difference was not large; 0.19.

**Table 4:** Differences in latent means between groups on the *Short Understanding of Substance Abuse Scale* (SUSS) for *N* = 640 participants^a^

	Professionals^b^	Patients	General public
Disease model beliefs	0.00	1.02 (0.10), *p* < 0.001	0.25 (0.09), *p* = 0.006
Psychosocial model beliefs	0.00	0.13 (0.09), *p* = 0.131	−0.19 (0.07), *p* = 0.006

Notes:

^a^Latent means are obtained from a Multigroup confirmatory factor analysis with cross-groups partial scalar invariance. The brackets show standard errors

^b^Reference group

Effect sizes are not directly computed in Mplus. In order to examine the magnitude of the difference in the latent mean scores, we calculated an effect size *d* for the latent mean differences following the procedure proposed by Hancock [37]. The effect size *d* for the patients versus the professionals was 1.15 for disease model beliefs and 0.23 for psychosocial model beliefs. For the general public group versus the professionals, the effect size *d* was 0.28 for disease model beliefs and 0.33 for psychosocial model beliefs. According to interpretative guidelines; 0.2, 0.5 and 0.8 are cut-off values for a small, medium and large effect size of latent means, this indicates a large effect size of patients versus the professionals on disease model beliefs and a small sized effect on the other differences between groups [37].

**Fig. 2:**
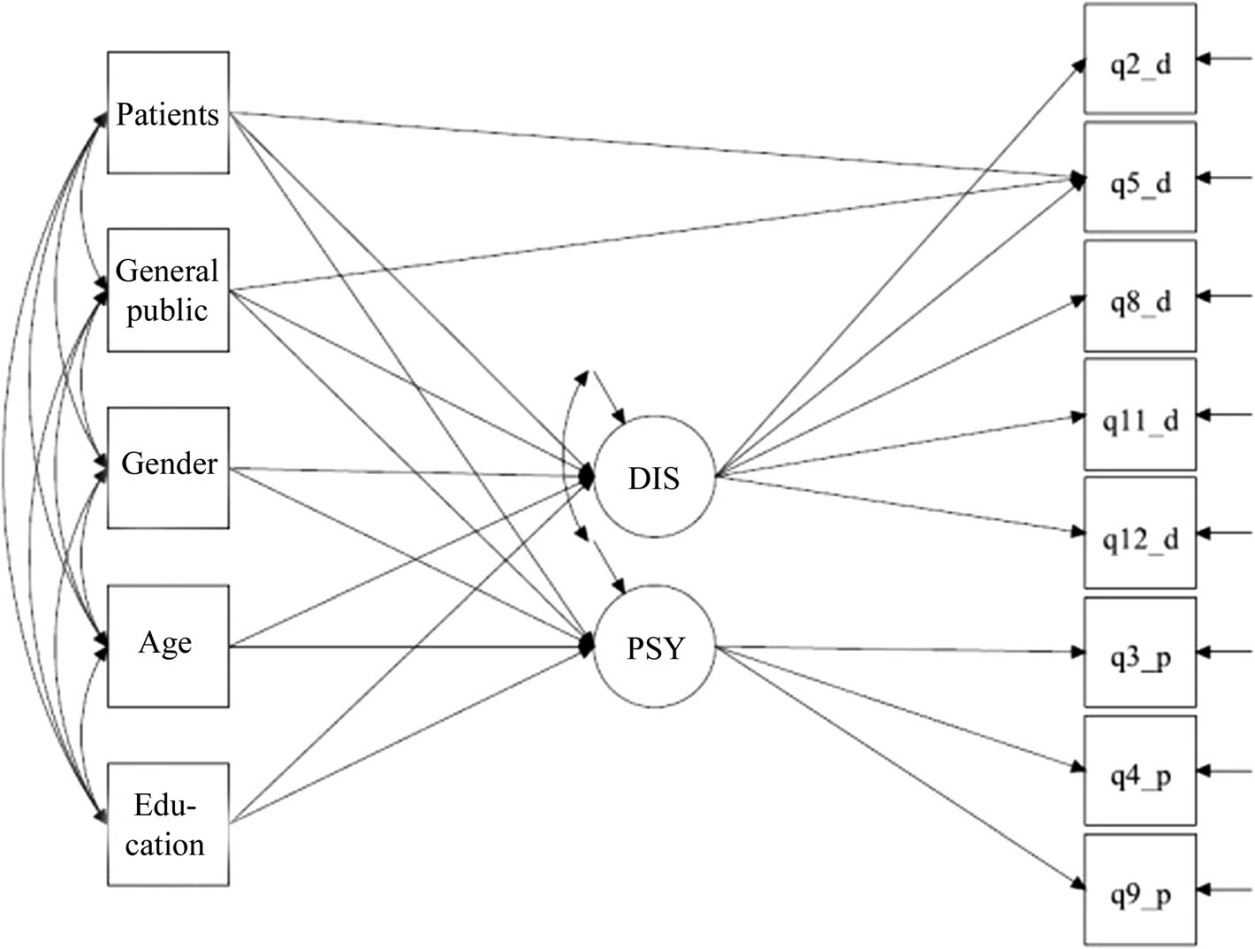
Multiple indicator multiple cause model of the *Short Understanding of Substance Abuse Scale* (SUSS), *N* = 640 participants. Notes: − DIS = disease model beliefs - PSY = psychosocial model beliefs - Education = length of education (years) - Q2_d, Q5_d, Q8_d, Q11_d, Q12_d = indicators for the disease model beliefs, see Table 1 for question wording - Q3_p, Q4_p, Q9_*p* = indicators for the psychosocial model beliefs, see Table 1 for question wording

Figure 2 shows the path diagram for the proposed MIMIC model tested with MPLUS. As the intercept of question 5 was freely estimated across groups in the MGCFA model, we allowed direct relationship between the dummy variables representing the groups and the item question 5. This relationship, termed differential item bias, reflects the fact that group membership has a direct effect on question 5 and not all the effect is mediated by the latent construct itself. Table 5 reports the estimates of latent mean difference obtained from the MIMIC model when controlled for gender, age and educational level. Compared to the results from the MGCFA model in Table 4, the estimated latent mean differences for psychosocial model beliefs changed only slightly and attained significance. However, the differences for the disease model beliefs reduced in size and the difference between the professionals and the general public group turned non-significant.

**Table 5:** Differences in latent means between groups on the *Short Understanding of Substance Abuse Scale* (SUSS) controlling for age, gender and education^a^

	Professionals^b^	Patients	General public
Disease model beliefs	0.00	0.67 (0.13), *p* < 0.001	0.13 (0.09), *p* = 0.156
Psychosocial model beliefs	0.00	0.19 (0.11), *p* = 0.07	−0.17 (0.07), *p* = 0.019

Notes:

^a^Differences in latent means are obtained from a multiple indicator multiple cause (MIMIC) model. The brackets show standard errors

^b^Reference group

The two constructs, disease and psychosocial model beliefs, were uncorrelated (*r* = −.01, *p* = 0.76). The two scales thus tap distinct and unrelated constructs.

## Discussion

This examination of the SUSS yielded a partial scalar invariant model. Hence, we were able to compare latent means between groups. In unadjusted comparisons, patients and the general public reported significantly higher endorsement of disease model beliefs than did professionals. The effect size of the difference between professionals and patients was large and persisted after controlling for possible confounders. In both unadjusted and adjusted analyses, the general public group but not the patient group scored lower than professionals on the psychosocial belief scale.

The findings in the present study indicate that the professionals were “out of step” with their patients as well as with the general public especially regarding their limited endorsement of the disease model. Prior SUSS studies have shown that this same pattern of beliefs hold in the U.S. for psychologists, but not for other addiction treatment professionals [8]. Both psychosocial and disease model belief systems can be said to have some support in the research literature in that interventions informed by each are comparably effective (e.g., 12-step facilitation counseling and CBT) [38]. Whether the patients or the professionals should change their beliefs in order to come to a shared view of SUD is a philosophical question as much as an empirical one, which observers will answer differently depending on whether they think health care professionals or those with experiential knowledge should be able to define illnesses [39].

In any event, what are the consequences of the different views in the communication between professionals and patients? If professionals are not aware of or do not appreciate such common understandings of addiction, their patients may feel misunderstood or alienated and drop out of care. The orientation of the professional should not be to prove that they are right and the client is wrong, but to accept that there are multiple useful ways to understand the experience of addiction. One of the important new trends in the health services in general is the focus on patient-centered care [40]. The overall idea is to promote active patient involvement in which patient and clinician communicate their expectations and preferences for treatment, and jointly agree upon the treatment to be implemented [41]. Differences in beliefs may be a challenge in achieving this in Norwegian addiction treatment. The likelihood of treatment goals being realized is generally assumed to be higher when patients and clinicians agree on them, and the professionals are responsible for establishing a common ground [42]. Being patient-centered may require clinicians be accepting of patients’ disease model beliefs even when the professionals do not share them.

As for the comparison between the professionals and the general public, the difference was in the expected direction when it came to the psychosocial model. Education often exposes the professional to learning theory, which would translate into greater endorsement of a psychosocial understanding of substance misuse [43]. Maybe for similar reasons, lay respondents had a higher support for the disease perspective than professionals, although the difference was not as large as that between the patients and the professionals. It may be tempting to dismiss the public’s views as a product of ignorance of the realities of addiction, but the view of the lay respondents concerning the disease model beliefs tended to draw in the same direction as those experiencing the disorder. It is possible that professional training in Norway actively “trains out” professionals from the most widespread understandings of addiction, whereas in other countries professional education more closely maps it. This is not in an absolute sense good or bad, except to the extent that misunderstandings and miscommunication between the public and professionals may lower support for treatment. However, we perceive that there is a strong support for SUD treatment initiatives and also support to pay taxes for it in the Norwegian society [44]. One might speculate that the prevalent endorsement of the disease model of SUD among the public actually paves the way for this support by legitimizing treatment as a medical activity.

The finding that the two constructs were uncorrelated means that a high endorsement of disease model beliefs does not exclude a high endorsement of psychosocial model beliefs and vice versa. Despite the emphasis that is sometimes placed on the alleged conflict between 12-step beliefs and psychosocial learning approaches [14], in reality many people integrate them smoothly. When examining the questions of the disease model beliefs, one can see that they are concerned with the need for continued abstinence, and as such, pertain to dependence as a chronic condition. The psychosocial model belief questions bear upon the development of the condition. Our findings indicate that strong endorsement of disease model beliefs does not necessarily mean that psychosocial factors that may have contributed to the condition are unrecognized. Conversely, when people have a high focus on the development of the condition and have high scorings on the psychosocial model belief scale, they may still recognize abstinence as a vital treatment goal [14].

As a direct comparison of these three groups has never been undertaken before, it is difficult to put our finding into an international perspective. One possible way to do it, would be to compare the sum scores of all the SUSS questions (see Table 1) with previous studies among professionals. The disease model belief scale was in the present study somewhere in-between previous findings from Switzerland and the U.S. The Norwegian professionals scored a mean of 15.8 (scale 0–28), whereas previous research showed that Swiss professionals scored 8.4 and U.S. professionals scored 18.5 [8, 15]. Norway is not at all dominated by the classic AA-influenced disease model in its addiction treatment services; less than 5 % of the addiction treatment centers employ a 12-step based treatment model [16]. Thus, it is unexpected that Norwegian professionals seemed to endorse disease model beliefs more than do Swiss personnel. This finding may reflect that Norwegian professionals still consider abstinence as the main treatment goal for patients with dependence, although a previous study found that the acceptance of non-abstinence as a treatment goal was widespread [45]. That study assessed agency policies and practices, though, and did not assess the attitudes of individual providers. Concerning the psychosocial model beliefs, the scores were quite similar among the countries (Swiss = 12.3, Norway = 13.0 and the U.S. = 13.2). We have not been able to find normative data from studies among patients.

### Methodological considerations

How generalizable are these results to Norway as a whole? Although slightly older (41 versus 38 years), the patient sample was similar to the detox patients (*N* = 564) of a previous regional multisite study [46]; they exhibited similar gender, ethnicity, major substance of abuse, and previous SUD treatment [21]. We have no reason to believe that the professionals of the surveyed health region differ from other regions in Norway. Thus, the findings of the present study were generalizable to other detox patients in the region and addiction professionals in Norway at large. However, as the patients were in-patients on a detoxification unit, the findings may not be generalized to patients with less severe SUDs. The general public survey was undertaken years later than in the other groups. We are not aware of any large-scaled public debates or changes in policy that may have contributed to temporal changes in the public opinion during this period. One could expect that the Facebook recruitment would bias the results by appealing more to younger, technology-saavy individuals, but this was not apparent in the data; the mean age was quite similar across groups. The general public sample was a convenience sample, and thus, the representativeness of this sample to the population at large is uncertain. Friendship with an author could possibly influence a participant’s beliefs by those of the author [47]. To avoid this social contagion bias as much as possible, the link to the online survey was not placed directly on the first author’s Facebook account; rather it was posted on different Facebook groups or sent to contacts of friends of the first author. Further, those of the author’s social contacts who work in the addiction or psychiatry fields could not have participated. However, as this data collection was anonymous, we cannot rule out that there may have been some overlap with the first author’s friends. The first application of a validated scale in a new country and the first ever examination of beliefs in the general population remain strengths of the study.

### Future research

Based on the existing questions in the SUSS, there is sparse information in the disease model construct on how the illness develops. Conversely, there is no information in the psychosocial model construct on how to cope with the illness. Thus, it would be interesting to expand this type of research and combine it with the model of helping and coping suggested by Brickman et al. [48]. These authors emphasize the necessity of distinguishing between attribution of responsibility for a problem (who is to blame), and attribution of responsibility for a solution to the problem (who is in control of the needed action to solve it), yielding a more differentiated framework. For example, the disease model construct is differentiated into two categories; the typical medical model, where neither the illness nor the treatment is the person’s responsibility, whereas in the compensatory model, people are not blamed for their problems, but are still held responsible for and expected to be actively involved in solving these problems. The latter is popularly articulated in the words of Jesse Jackson; “You are not responsible for being down, but you are responsible for getting up!” This is in line with the philosophy of the AA program, in which the individual is not blamed for having the disease, but is certainly held responsible for taking action steps to maintaining remission [49, 50]. Brickman et al. state that a wrong choice of model may undermine both an effective helping and a coping strategy. A combination of the SUSS and Brickman et als’ models would allow an examination of the conception of addiction in a more differentiated framework.

## Conclusions

As was found in the Swiss study, the disease and psychosocial model subscales of the SUSS worked well in a Norwegian sample after some amendments to the constituent items. This suggests both that the SUSS has broad cultural application but at the same time should be tested and validated in each culture or subculture in which it is employed. More cross-cultural tests of the scale – which is available free of charge to researchers and clinicians – would be highly desirable.

Substantively, the study found that addiction treatment professionals have different understandings of substance use disorder than patients and the general public. Care must be taken that this does not result in miscommunication or poor relationships that could compromise the quality of the therapeutic alliance.

## References

[1] Humphreys K, Noke JM, Moos RH (1996). Recovering substance abuse staff members’ beliefs about addiction. J Subst Abuse Treat.

[2] Moyers TB, Miller WR (1993). Therapists’ conceptualizations of alcoholism: Measurement and implications for treatment decisions. Psychol Addict Behav.

[3] Ouimette P, Humphreys K, Moos RH, Finney JW, Cronkite R, Federman B (2001). Self-help group participation among substance use disorder patients with posttraumatic stress disorder. J Subst Abuse Treat.

[4] Sheehan T, Owen P, McCrady B, Epstein E (1999). The disease model. Addictions - A comprehensive guidebook.

[5] Owen P, Marlatt GA (2001). Should abstinence be the goal for alcohol treatment? Affirmative viewpoint. Am J Addict.

[6] Smith BD, Manfredo IT (2011). Frontline counselors in organizational contexts: a study of treatment practices in community settings. J Subst Abuse Treat.

[7] Bean-Bayog M, McCrady B, Miller WR (1993). AA processes and Change: How does it work?. Research on alcoholics anonymous.

[8] Humphreys K, Greenbaum MA, Noke JM, Finney JW (1996). Reliability, validity, and normative data for a short version of the understanding of alcoholism scale. Psychol Addict Behav.

[9] Cook CC (1988). The Minnesota Model in the management of drug and alcohol dependency: miracle, method or myth? Part I. The philosophy and the programme. Br J Addict.

[10] Anderson DJ, McGovern JP, DuPont RL (1999). The origins of the Minnesota model of addiction treatment--a first person account. J Addict Dis.

[11] Magura S (2007). The relationship between substance user treatment and 12-step fellowships: current knowledge and research questions. Subst Use Misuse.

[12] Leshner AI (1997). Addiction is a brain disease, and it matters. Science.

[13] Meurk C, Carter A, Partridge B, Lucke J, Hall W (2014). How is acceptance of the brain disease model of addiction related to Australians inverted question mark attitudes towards addicted individuals and treatments for addiction?. BMC Psychiatry.

[14] Burke AC, Clapp JD (1997). Ideology and social work practice in substance abuse settings. Soc Work.

[15] Moggi F, Giovanoli A, Sutter M, Humphreys K (2005). Validity and reliability of the German version of the short understanding of substance abuse scale. Eur Addict Res.

[16] Vederhus JK, Kristensen O, Laudet A, Clausen T (2009). Attitudes towards 12-step groups and referral practices in a 12-step naive treatment culture; a survey of addiction professionals in Norway. BMC Health Serv Res.

[17] Vederhus JK, Laudet A, Kristensen O, Clausen T (2010). Obstacles to 12-step group participation as seen by addiction professionals: comparing Norway to the United States. J Subst Abuse Treat.

[18] Steinmetz H, Schmidt P, Tina-Booh A, Wieczorek S, Schwartz SH (2009). Testing measurement invariance using multigroup CFA: Differences between educational groups in human values measurement. Qual Quant.

[19] Byrne BM, Shavelson RJ, Muthen B (1989). Testing for the equivalence of factor covariance and mean structures: The issue of partial measurement invariance. Psychol Bull.

[20] Gordoni G, Schmidt P, Gordoni Y (2011). Measurement Invariance across Face-to-Face and Telephone Modes: The Case of Minority-Status Collectivistic-oriented Groups. Int J Public Opin Res.

[21] Vederhus JK, Timko C, Kristensen O, Hjemdahl B, Clausen T (2014). Motivational intervention to enhance post-detoxification 12-Step group affiliation: a randomized controlled trial. Addiction.

[22] Vederhus JK, Timko C, Kristensen O, Clausen T (2011). The courage to change: patient perceptions of 12-Step fellowships. BMC Health Serv Res.

[23] Ministry of Justice and Public Security. Personal Data Act. Oslo: Ministry of Justice and Public Security; 2001. Cited 12.02.2016 at https://www.datatilsynet.no/English/Regulations/Personal-Data-Act-/.

[24] Beaton DE, Bombardier C, Guillemin F, Ferraz MB (2000). Guidelines for the process of cross-cultural adaptation of self-report measures. Spine.

[25] Brown TA (2006). Confirmatory factor analysis for applied research.

[26] Gjersing L, Caplehorn JR, Clausen T (2010). Cross-cultural adaptation of research instruments: language, setting, time and statistical considerations. BMC Med Res Methodol.

[27] Bollen KA (1989). Structural equations with latent variables.

[28] Davidov E, Meuleman B, Cieciuch J, Schmidt P, Billiet J (2014). Measurement equivalence in cross-national research. Annu Rev Sociol.

[29] Muthén LK, Muthén BO (2010). Mplus. Statistical analysis with latent variables - user’s guide.

[30] Stevens JP (2009). Applied multivariate statistics for the social sciences.

[31] Hu L-t, Bentler PM (1999). Cutoff criteria for fit indexes in covariance structure analysis: Conventional criteria versus new alternatives. Struct Equ Modeling.

[32] Little TD (2013). Longitudinal structural equation modeling.

[33] Steenkamp JEM, Baumgartner H (1998). Assessing measurement invariance in cross-national consumer research. J Consum Res.

[34] Cheung GW, Rensvold RB (2002). Evaluating goodness-of-fit indexes for testing measurement invariance. Struct Equ Modeling.

[35] Kline RB (2011). Principles and practice of structural equation modeling.

[36] Thompson MS, Green SB, Hancock GR, Mueller RO (2013). Evaluating between-group diffrences in latent variable means. Structural equation modeling - a second course.

[37] Hancock GR (2001). Effect size, power, and sample size determination for structured means modeling and MIMIC approaches to between-groups hypothesis testing of means on a single latent construct. Psychometrika.

[38] Project MATCH Research Group (1998). Matching alcoholism treatments to client heterogeneity: Project MATCH three-year drinking outcomes. Alcohol Clin Exp Res.

[39] deBronkart D (2015). From patient centred to people powered: autonomy on the rise. BMJ.

[40] Stevenson FA, Barry CA, Britten N, Barber N, Bradley CP (2000). Doctor-patient communication about drugs: the evidence for shared decision making. Soc Sci Med.

[41] Joosten EA, de Jong CA, de Weert-van Oene GH, Sensky T, van der Staak CP (2009). Shared decision-making reduces drug use and psychiatric severity in substance-dependent patients. Psychother Psychosom.

[42] Fakhoury WK, Priebe S, Quraishi M (2005). Goals of new long-stay patients in supported housing: a UK study. Int J Soc Psychiatry.

[43] Koski-Jannes A, Hirschovits-Gerz T, Pennonen M (2012). Population, professional, and client support for different models of managing addictive behaviors. Subst Use Misuse.

[44] Meld.St. 30(2011–2012): Se meg! : en helhetlig rusmiddelpolitikk : alkohol - narkotika - doping. Oslo: Helse- og omsorgsdepartementet,.

[45] Duckert F, Duckert F, Koski-Jännes A, Rönnberg S (1989). Controlled drinking’ - A complicated and contradictory field. Perspectives on controlled drinking.

[46] Hobbesland Å (2006). Evaluation of detoxification treatment in six treatment centers in the Southern Health Region.

[47] Christakis NA, Fowler JH (2013). Social contagion theory: examining dynamic social networks and human behavior. Stat Med.

[48] Brickman P, Rabinowitz VC, Karuza J, Coates D, Cohn E, Kidder L (1982). Models of helping and coping. Am Psychol.

[49] Gossop M, Gossop M (2003). Narcotics anonymous, twelve-step programmes, and rehabilitation treatments. Drug addiction and its treatment.

[50] Morojele NK, Stephenson GM (1992). The Minnesota Model in the treatment of addictions: A social psychological assessment of changes in beliefs and attributions. J Community Appl Soc.

